# Supplementary material for: Specialized Tfh cell subsets driving type-1 and type-2 humoral responses in lymphoid tissue
Source: Cell Discov. 2024 Jun 4;10:64. doi: 10.1038/s41421-024-00681-0 (PMC11150427; doi:10.1038/s41421-024-00681-0)
Supplement: Supplementary file 1 — Supplementary Information [file 41421_2024_681_MOESM1_ESM.pdf]

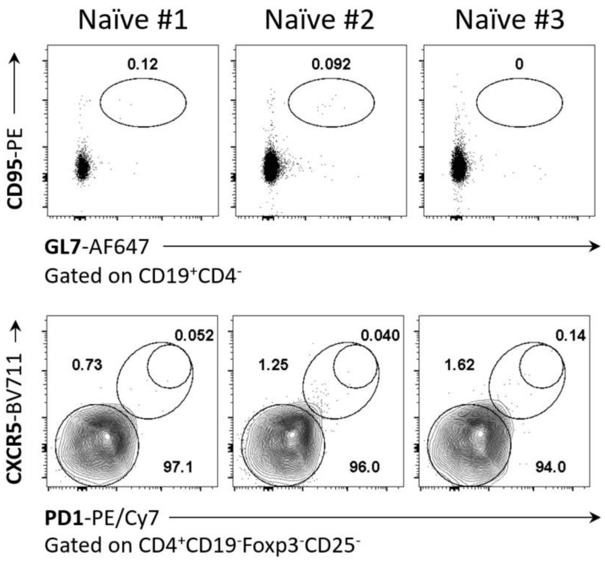

Supplementary Figure 1: Popliteal lymph nodes from non-immunized mice are devoid of GC and Tfh cells. Representative dotplots of cells from popliteal lymph nodes of three non-immunized C57Bl/6 mice (see Figure 1c as a reference). We could not identify GC B cells ($CD95^{+}GL7^{+}$), nor Tfh cells ($CXCR5^{+}PD\text{-}1^{+}$).

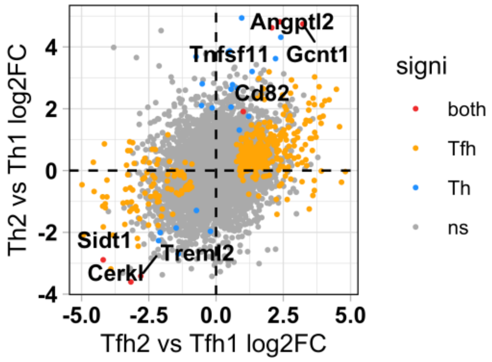

Supplementary Figure 2: Gene expression coherence between Tfh and Th populations. Representation of genes that are coherently overexpressed in both Tfh1 and Th1 cells, and Tfh2 and Th2 cells (red). The x-y plot also represents the genes that are specifically overexpressed in Tfh1 or Tfh2 cells (but not the Th populations, in orange); and overexpressed in Th cells but not on the Tfh populations (blue). Genes represented in grey are not significantly different between populations.

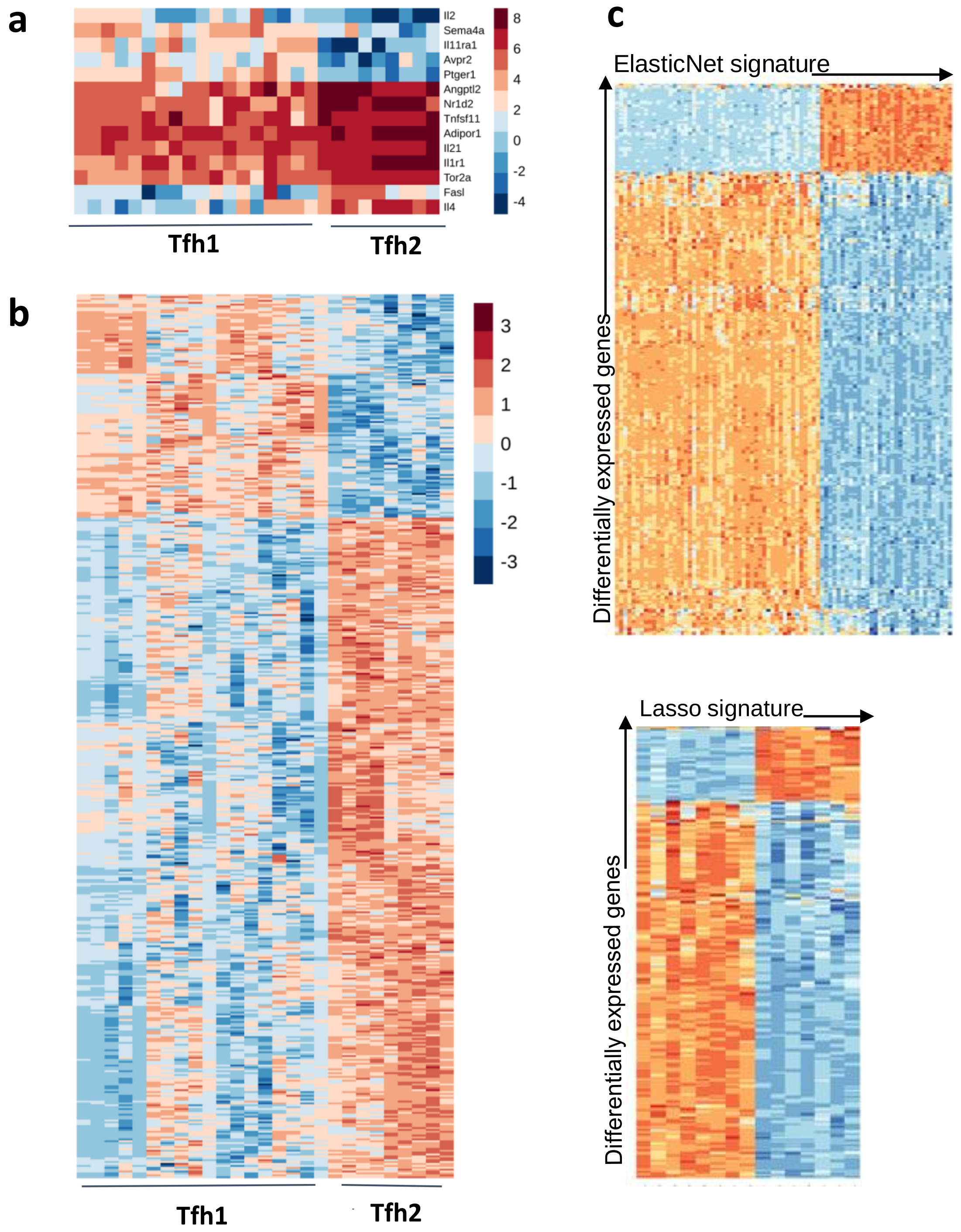

Supplementary Figure 3: Differential Gene expression of Tfh1 and Tfh2 subsets. **(a)** Unscaled heatmap of genes matched with immune gene list in Tfh2 vs Tfh1 comparison. The plot shows that while *Il21*, *Tnfsf11*, and *Angptl2* are differentially expressed between the two subpopulations, these genes are expressed in all Tfh samples consistently. **(b)** Heatmap of significantly differentially expressed genes between Tfh1 and Tfh2 cell subsets from both mouse strains. Genes with adjusted p values < 0.05 were considered significant. Values are scaled for each gene across all samples. The heatmap shows inconsistent patterns of expression from all samples of the same category (Tfh1, Tfh2) (**c**) Correlation of the transcriptome signature from Lasso (top) and ElasticNet (bottom) against the differentially expressed genes.

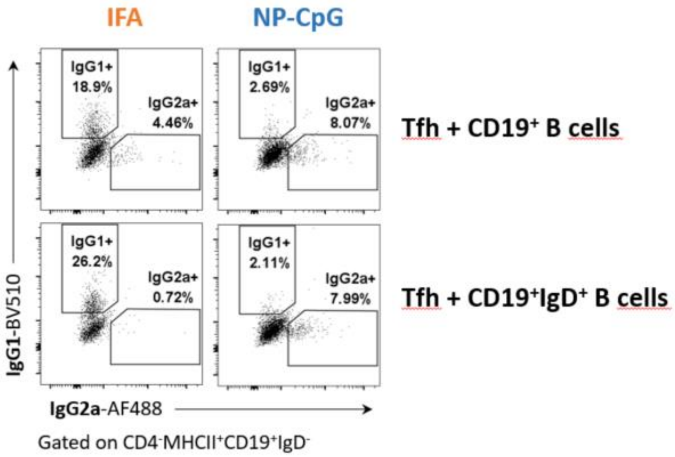

Supplementary Figure 4: Functional assessment of Tfh1 and Tfh2 cells. C57BL/6 mice were immunized in the footpad with OVA emulsified in IFA (IFA) or incorporated with CpG in nanoparticles (NP-CpG). On day 11, Tfh ($CD4^+CD25^-CXCR5^+PD1^+$), the global B cells ($CD19^+CD4^-$), or naïve B cells ($CD19^+CD4^-IgD^+$) were isolated from draining LNs by flow cytometry and co-cultured. $IgG2a^+$ and $IgG1^+$ isotype-switched B cells were assessed at the end of the co-cultures. Similar results were obtained in cultures with the entire B cell population or with naïve B cells.

a

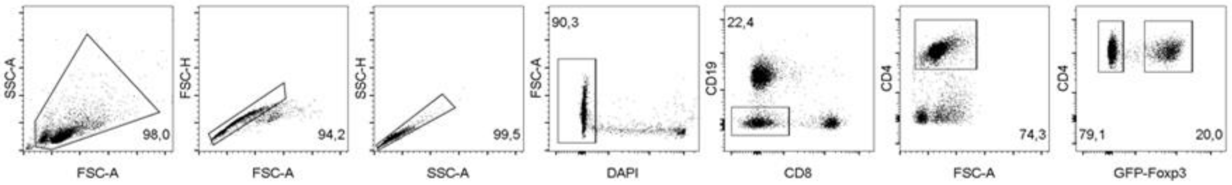

b

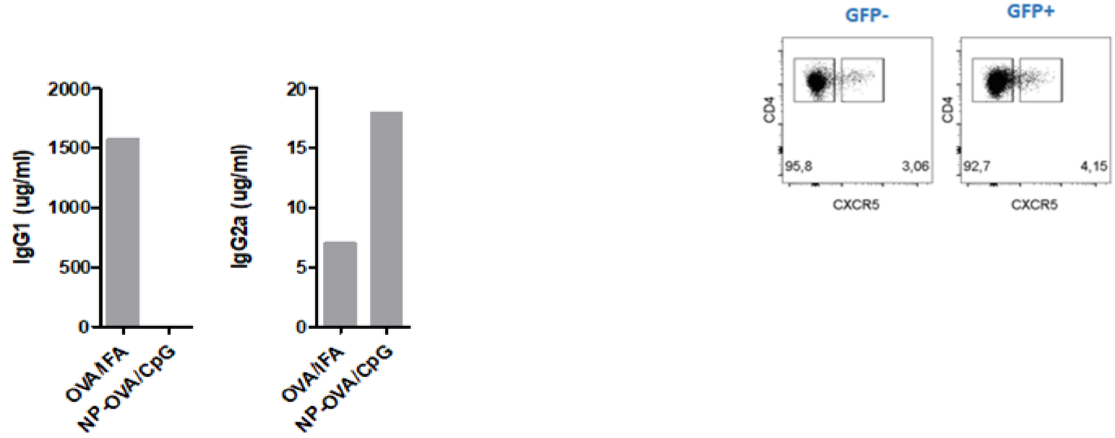

Supplementary Figure 5. Single cell RNA-seq for Tfh and Treg cells. (a) Gating strategy used for cell sorting of Tfh and Treg cells after 11 days of immunization. **(b)** ELISA quantification of OVA IgG1 and IgG2a in the serum of immunized mice.

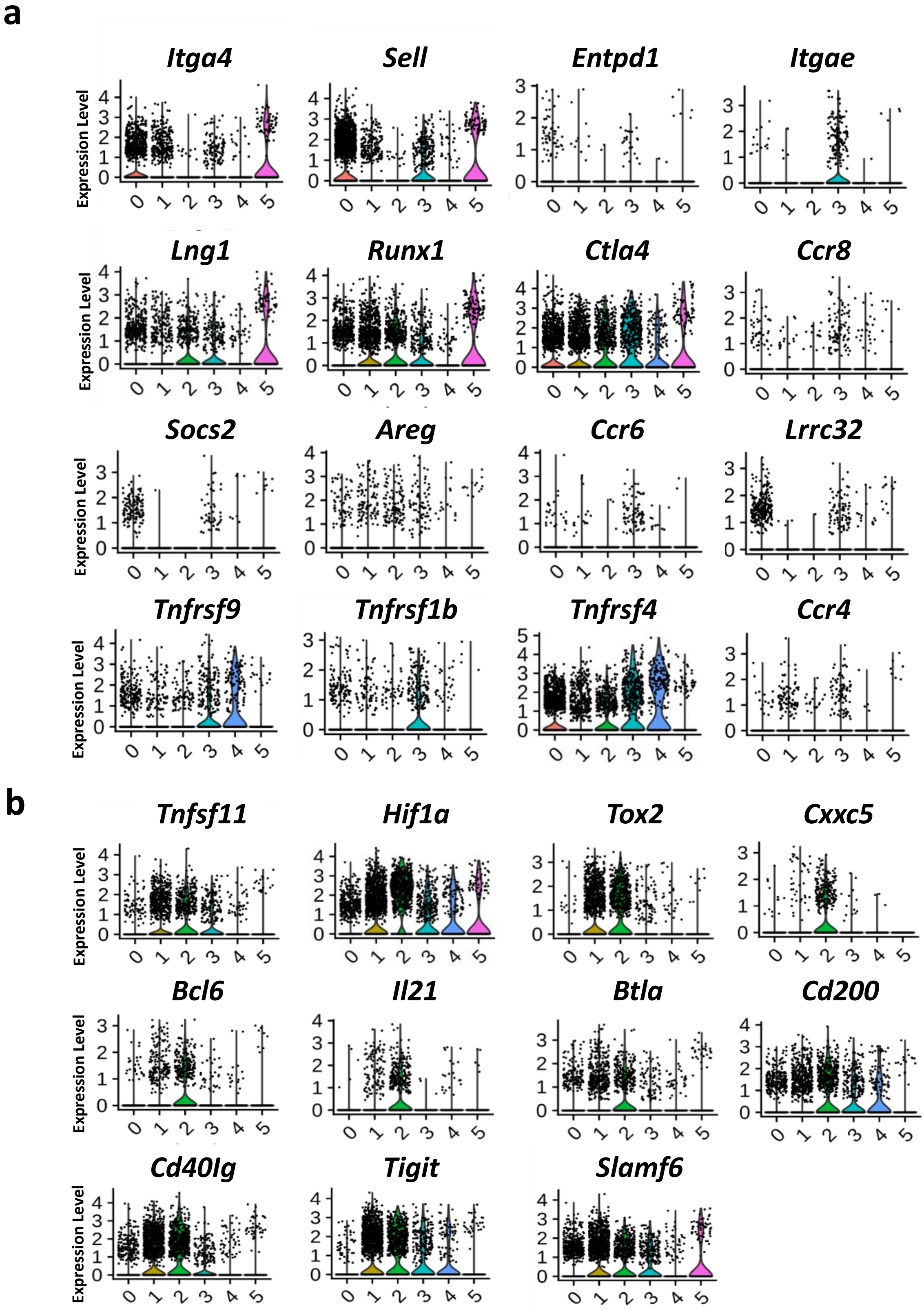

Supplementary Figure 6. Tfh an Treg markers. Violin plots of a selection of transcripts associated to **(a)** Treg and **(b)** Tfh cells, describing the heterogeneity of these cells in different clusters.

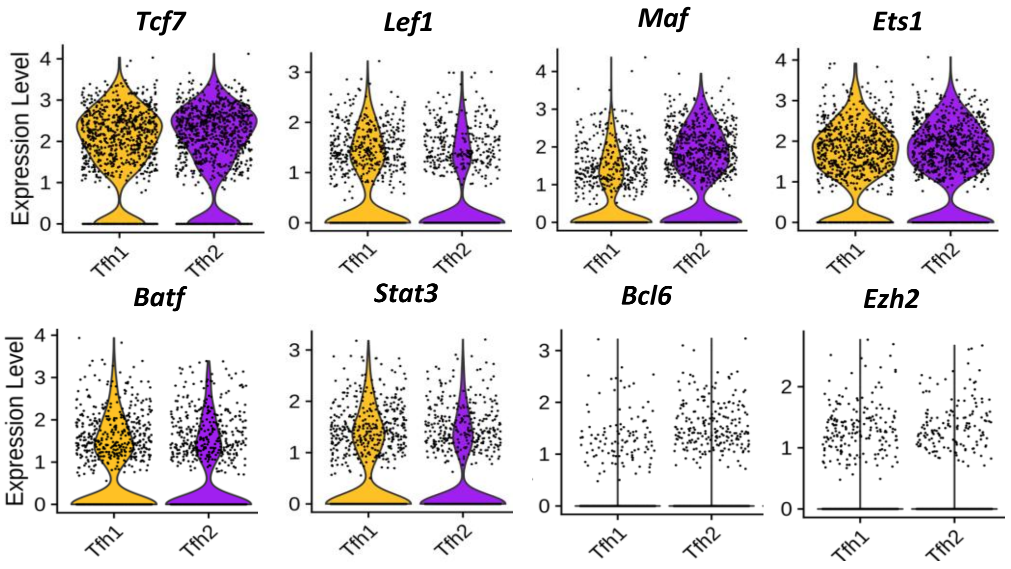

Supplementary Figure 7. Transcription factors in Tfh1 and Tfh2 cells . Violin plots showing expression of known transcription factors in both Tfh1 and Tfh2 clusters. The plots show comparable expression in both subsets.

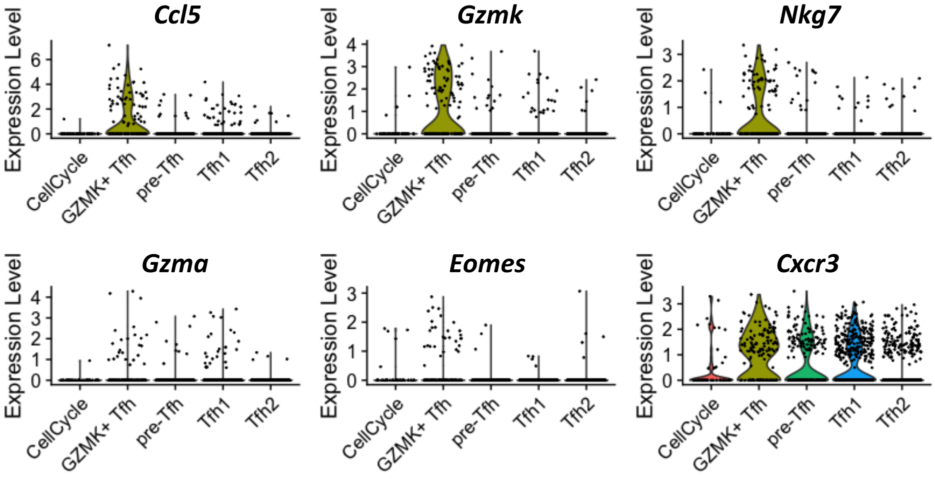

Supplementary Figure 8. Expression profile of transcripts associated with the Gzmk⁺ Tfh cell population. Expression profile of differential transcripts that characterize the Gzmk⁺ Tfh cell population across all subsets of Tfh cells identified as represented in Figure 8e.

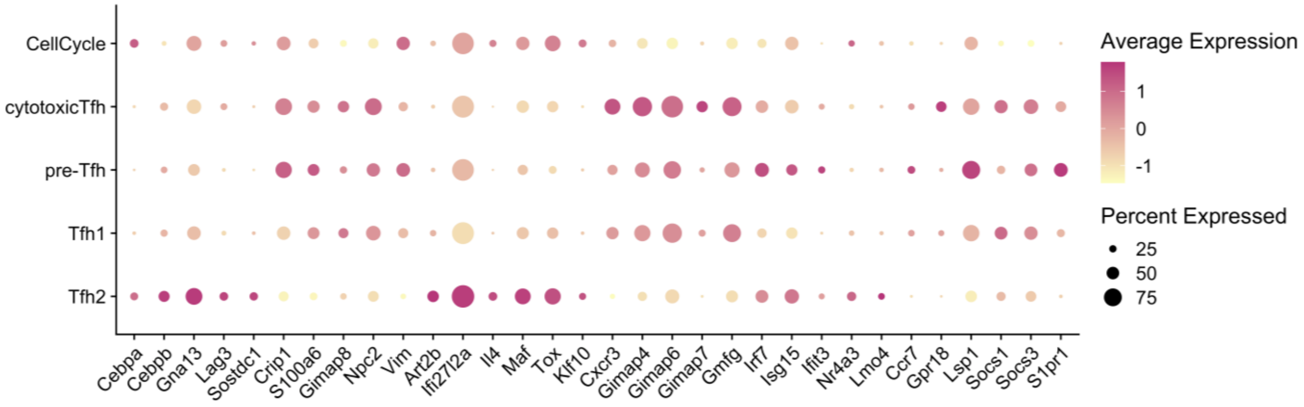

Supplementary Figure 9. Expression profile of transcripts associated with Tfh1 and Tfh2 populations. Expression of Tfh1 and Tfh2 genes across all subsets of Tfh cells identified as represented in Figure 7f.

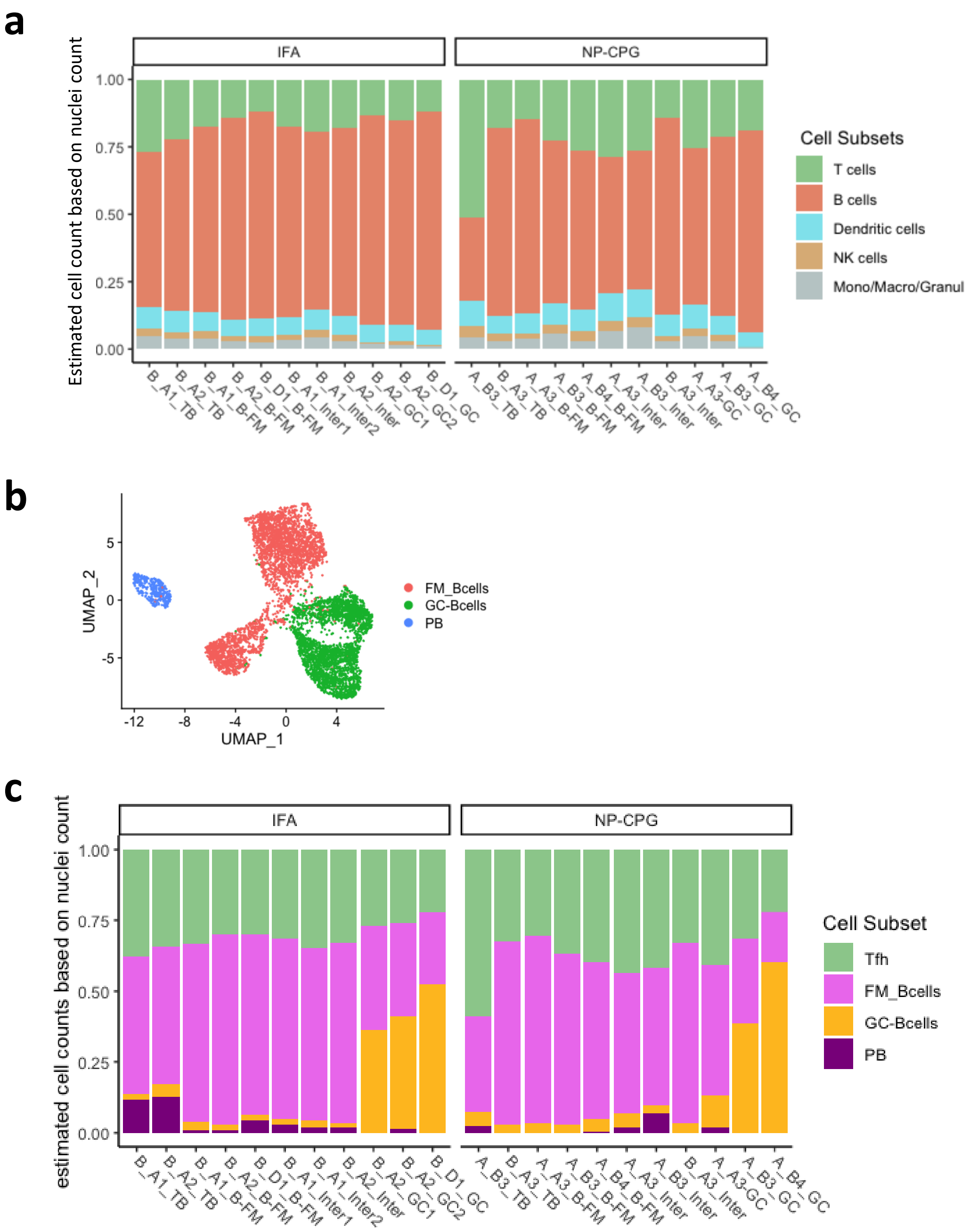

Supplementary Figure 10. Spatial deconvolution of ROIs. **(a)** Spatial deconvolution of ROIs using GEOMx mouse spleen profile data for each of the immunizations. **(b)** UMAP of B cells from publicly available B cell dataset (GSE189819) used for deconvolution. In this dataset, the B cells were classified as having a germinal centre (GC), follicular mantle (FM), or plasmablast (PB) transcriptional profile. **(c)** Spatial deconvolution of ROIs using a profile matrix generated using single cell Tfh and the public B cell datasets represented above.

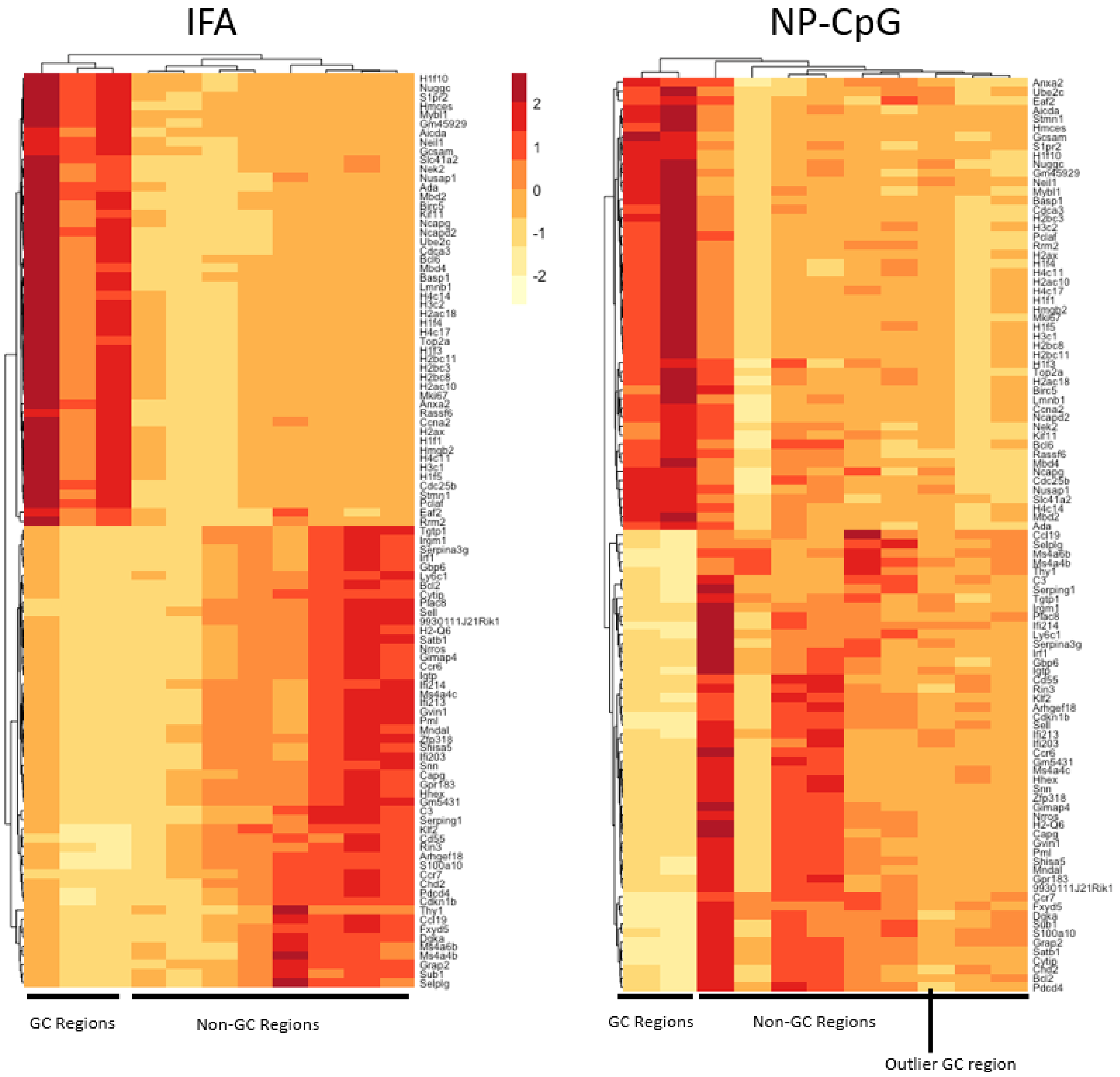

Supplementary Figure 11. Top transcripts that discriminate GC and non-GC regions defined with Geo-Mx. When comparing the transcripts associated with the different areas of interest defined with Geo-Mx we found a clear distinction between GC regions and other regions, as represented in the PCA in Fig. 8I. The PC1 discriminated GC and non-GC (21% variance). The heatmaps show the top 50 transcripts that explain PC1 in type-2 conditions (IFA) and type-1 (NP-CpG).

Cell Type	Response Type	Mouse Strain	Immunization	Batch	No of Samples	Sequence Type	Read Length	Average no of cells
Tfh	1	C57B6J	CpG	2	5	Single-end	75	564
Tfh	1	C57B6J	NP-CpG	4	4	Single-end	85	1036
Tfh	2	C57B6J	IFA	2	5	Single-end	75	5000
Th	1	C57B6J	CpG	2	5	Single-end	75	4656
Th	1	C57B6J	NP-CpG	4	4	Single-end	85	1357
Th	2	C57B6J	IFA	2	5	Single-end	75	5000
Tfh	1	BALBC	CpG	1	5	Paired-end	100	70
Tfh	1	BALBC	NP-CpG	3	4	Single-end	85	1011
Tfh	2	BALBC	IFA	1	4	Paired-end	100	350
Th	1	BALBC	CpG	1	5	Paired-end	100	187
Th	1	BALBC	NP-CpG	3	4	Single-end	85	4114
Th	2	BALBC	IFA	1	4	Paired-end	100	275

Supplementary Table 1. Summary of all samples sequenced for each immunization and from the two strains.

**Category**	**Immunization**	**No of samples**	**Day of collection**	**Tissue**
Training Data	IFA: type-2	9	11	Popliteal Lymph Node
Training Data	NP-CpG: type-1	8	11	Popliteal Lymph Node
Training Data	CpG: type-1	10	11	Popliteal Lymph Node
Test Data	Helminth	6	8	Spleen
Test Data	LCMV	4	8, 10	Spleen

Supplementary Table 2. Samples used to generate the transcriptional signatures.

**Sample Name**	**Infection**	**ElasticNet classification**	**Lasso classification**
SRR2225034	Helminth	2	2
SRR2225035	Helminth	2	2
SRR2225036	Helminth	2	2
SRR2225037	Helminth	2	2
SRR2225038	Helminth	2	2
SRR2225039	Helminth	2	2
SRR3214186	LCMV	1	1
SRR3214190	LCMV	1	1
SRR6203712	LCMV	1	1
SRR6203713	LCMV	1	1

Supplementary Table 3. Public datasets used for validation, and results of elasticnet and lasso classifiers in classifying each sample as belonging to either the type-1 or type-2 category.

Table S4: Antibody details					
**Target**	Clone	Fluorophore	Source	Code	Concentration
**CD4**	RM4-5	BV421	BioLegend	100543	1:100
**GL7**	GL7	AF488	BioLegend	144612	1:100
**IgD**	11-26c.2a	NIR685	BioLegend	405749	1:200

Supplementary Table 4. Antibodies used for histology. These antibodies are related to the image in Figure 7c.
